# The Impact of Medical Students’ Individual Teaching Format Choice on the Learning Outcome Related to Clinical Reasoning

**DOI:** 10.2196/13386

**Published:** 2019-07-22

**Authors:** Nikolai Schuelper, Sascha Ludwig, Sven Anders, Tobias Raupach

**Affiliations:** 1 Department of Haematology and Medical Oncology University Medical Centre Göttingen Göttingen Germany; 2 Department of Anaesthesiology University Medical Centre Göttingen Göttingen Germany; 3 Department of Legal Medicine University Medical Centre Hamburg-Eppendorf Hamburg Germany; 4 Division of Medical Education Research and Curriculum Development Study Deanery of University Medical Centre Göttingen Göttingen Germany

**Keywords:** undergraduate medical education, case histories

## Abstract

**Background:**

Repeated formative assessments using key feature questions have been shown to enhance clinical reasoning. Key feature questions augmented by videos presenting clinical vignettes may be more effective than text-based questions, especially in a setting where medical students are free to choose the format they would like to work with. This study investigated learning outcomes related to clinical reasoning in students using video- or text-based key feature questions according to their individual preferences.

**Objective:**

The aim of this study was to test the hypothesis that repeated exposure to video-based key feature questions enhances clinical reasoning to a greater extent than repeated exposure to text-based key feature questions if students are allowed to choose between those different formats on their own.

**Methods:**

In this monocentric, prospective, nonrandomized trial, fourth-year medical students attended 12 computer-based case seminars during which they worked on case histories containing key feature questions. Cases were available in a text- and a video-based format. Students chose their preferred presentation format at the beginning of each case seminar. Student performance in key feature questions was assessed in formative entry, exit, and retention exams and was analyzed with regard to preceding exposure to video- or text-based case histories.

**Results:**

Of 102 eligible students, 75 provided written consent and complete data at all study exams (response rate=73.5%). A majority of students (n=52) predominantly chose the text-based format. Compared with these, students preferring the video-based format achieved a nonsignificantly higher score in the exit exam (mean 76.2% [SD 12.6] vs 70.0% [SD 19.0]; *P*=.15) and a significantly higher score in the retention exam (mean 75.3% [SD 16.6] vs 63.4% [SD 20.3]; *P*=.02). The effect was independent of the video- or text-based presentation format, which was set as default in the respective exams.

**Conclusions:**

Despite students’ overall preference for text-based case histories, the learning outcome with regard to clinical reasoning was higher in students with higher exposure to video-based items. Time-on-task is one conceivable explanation for these effects as working with video-based items was more time-consuming. The baseline performance levels of students do not account for the results as the preceding summative exam results were comparable across the 2 groups. Given that a substantial number of students chose a presentation format that was less effective, students might need to be briefed about the beneficial effects of using video-based case histories to be able to make informed choices about their study methods.

## Introduction

### Teaching Clinical Reasoning

One of the most challenging aims in undergraduate medical education is to teach students about how to arrive at a correct diagnosis and to initiate adequate therapeutic steps. Even for experienced physicians, clinical decision making is a critical aspect of their performance and different theories trying to elucidate the underlying cognitive mechanisms have been put forward [1]. Clinical reasoning reflects the involved aspects for decision making in the clinical context, and case-based learning turned out to be both effective for teaching clinical reasoning and is preferred by undergraduate medical students [2,3]. Among other assessment formats, key feature questions can be used to measure student performance in this particular area of expertise [4-6]. However, this type of assessment may not only be used to serve a summative purpose but also be used in a formative manner, taking advantage of the so-called direct testing effect [7]. Research published in the past 10 years supports the hypothesis that repeated testing enhances long-term retention of knowledge [8], skills [9], and—perhaps most importantly—the clinical application of knowledge [10]. We recently reported superior long-term retention of clinical reasoning performance in students who had repeatedly been exposed to formative key feature questions compared with students who had restudied the same content without being prompted to answer questions [11]. In that study, all study-related material was presented in written form. After 9 months, students scored significantly higher on intervention items trained with key feature questions compared with control items (mean 56.0% [SD 25.8] vs 48.8% [SD 24.7]; *P*<.001). In a further study comparing key feature cases with text-based case histories with video-based ones, these results were confirmed in a postintervention exam (mean 76.2% [SD 19.4] vs 72.4% [SD 19.1], *P*=.03) but not in a retention exam 9 months later (mean 69.2% [SD 20.2] vs 66.4% [SD 20.3], *P*=.11) [12].

### Presenting Formats

Case histories can be presented in different formats including text-based and video-based displays or even in a simulated clinical setting using standardized patients. It might be hypothesized that greater authenticity of the learning material entails more favorable learning outcomes. In contrast, a prospective, randomized study with 133 students did not yield any significant differences between those 3 presenting formats with regard to improvement of clinical reasoning performance [13]. Another study with 256 students showed preference for video cases versus paper cases arguing that videos preserve the original language, avoid depersonalization of patients, and facilitate direct observation of clinical consultations [14]. Despite the reported preference for video-based case presentations in a study nested in a problem-based learning setting, the same study showed that the use of videos might be associated with a reduction of the depth of thinking by analyzing 5224 transcripted student utterances by a blinded coder [15]. Conversely, an analysis of student critical thinking skills following exposure to different case modalities suggested that video-based material was particularly effective in fostering these skills [16]. Thus, the available evidence on the effectiveness of video-based instructional material for the training of clinical reasoning is equivocal.

### Learning Styles

One approach to understanding these conflicting data is the concept of *learning styles*, according to which characteristics of the way students learn predict the extent to which an individual student will benefit from specific teaching modalities [17]. Despite an ongoing debate on the usefulness of this approach [18], this concept is still underlying a considerable number of medical education research projects. Some of these studies refer to a model that distinguishes between different learning strategies, that is, visual, auditory, read and write, and kinesthetic [19]. In one study, individual learning styles of 62 applicants to general surgery were analyzed with respect to previous exam performance. Most applicants had a multimodal learning style, but aural and visual preferences were associated with significantly higher United States Medical Licensing Examination scores compared with read and write and kinesthetic preferences [20]. Owing to the lack of data supporting the idea that matching learning activities to individual learning styles does in fact lead to better learning outcomes, most intervention studies in the field of medical education did not assess the learning style, let alone account for it in their main analyses. At the same time, letting students choose their preferred learning modality (regardless of the *learning style*) may impact on the learning outcome, and this hypothesis has rarely been tested [13,21,22].

In summary, the available evidence supports the repetitive use of case-based key feature questions for teaching clinical reasoning. Furthermore, limited data indicate that medical students have individual preferences with regard to teaching modalities and that a higher degree of the authenticity of case presentations might foster the learning outcome in some students. However, it is unclear who will benefit most from using rich media and whether students are capable of identifying the method that works best for them. This study was designed to test the hypothesis that repeated exposure to video-based key feature questions enhances clinical reasoning to a greater extent than repeated exposure to text-based key feature questions if students are allowed to choose between those different formats on their own.

## Methods

### Study Design

This monocentric, prospective, nonrandomized intervention study investigated the impact of letting students choose their preferred learning format on the learning outcome with regard to clinical reasoning. The study consisted of a 3-month intervention phase followed by a nonintervention phase of 4 months. During the intervention phase, students attended 45-min weekly computer-based seminars (*electronic case seminars* [ECSs]) during which they worked on predefined patient case histories that were aligned to the learning objectives addressed in concurrent curricular teaching sessions. In the first and final weeks of the intervention phase as well as in the retention exam, students took identical formative key feature examinations.

**Figure 1:**
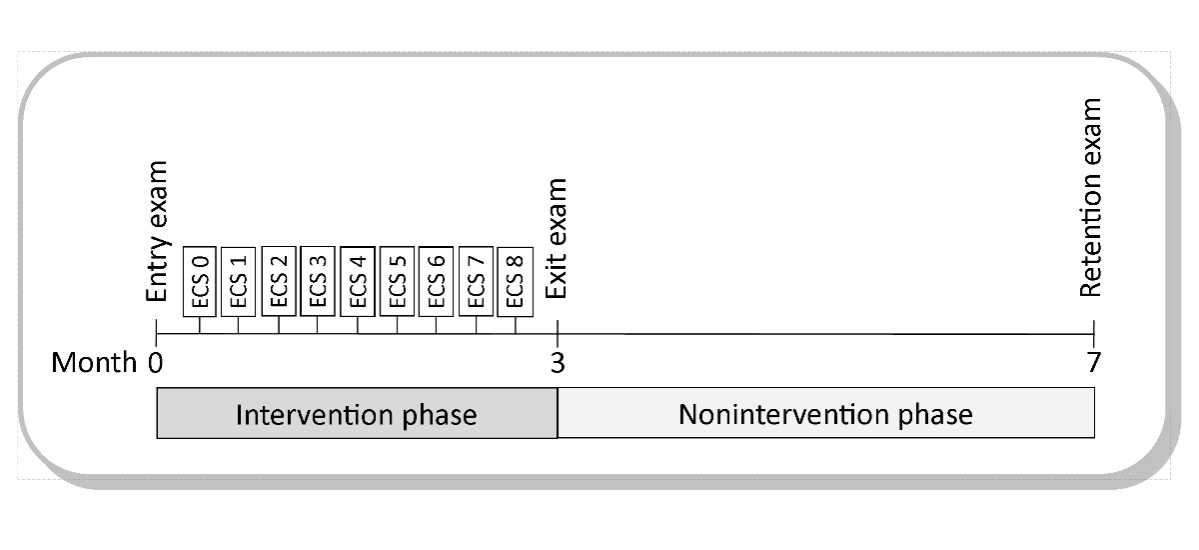
Timeline of study design and assessments. After 1 electronic case seminar (ECS) introducing text- and video-based case presentations (ECS 0), 8 weekly intervention ECSs with the free choice of learning format were conducted (ECS 1-8).

Students sat the unannounced retention exam following the 4-month nonintervention phase (see Figure 1).

All patient case histories were available in a text-based and video-based format (eg, Multimedia Appendix 1). During the first ECS, 4 cases were presented, 2 of which were video-based whereas the other 2 were text-based. Following this, students had the free choice of attending the learning format they preferred at the beginning of each ECS. In the entry, exit, and retention exam, an equal number of items were presented in a text- and video-based format. ECS attendance was mandatory for students enrolled in general medicine teaching modules of the fourth year.

### Student Recruitment and Ethics Approval

Fourth-year medical students at Göttingen Medical School were informed about the study 4 weeks ahead via email and during the first lecture of term. Students enrolled in all modules in winter term 2015 were eligible for study participation. The study was approved by the local ethics committee (*Ethik-Kommission der Universitätsmedizin Göttingen*, application number 10/12/15), and all participants provided written consent.

### Study Procedure

A total of 31 case histories were selected to be presented in the ECSs. All of these had been piloted and used in a previous research project [11]. Learning objectives and the content of cases were identical regardless of the video- or text-based presentation format. Patient case histories were broken up into 5 to 8 sections with key feature questions at the end of each section. All items that were used in the entry, exit, and retention exam occurred in 2 different ECSs during the intervention phase. Patient case histories differed regarding the particular story, but the key feature items were identical. During the intervention phase, each of the 9 ECSs consisted of 3 case histories with 5 key feature questions each. Thus, students answered a total of 135 original key feature questions addressing specific learning objectives during the 9 ECSs between the entry and exit exam. The entry, exit, and retention exam were made up of 4 case histories with a total number of 28 items, 14 of which were text based with the other 14 being presented as videos. Notably, for the 3 exams, the presenting format was set as default. As corresponding learning objectives to those 28 intervention items were taught twice during the intervention phase, and students had the choice between 2 different teaching formats at each time; there were 4 possible ways any one student could learn any of the 28 intervention items during the ECSs: text-text (sequence #1), text-video (sequence #2), video-text (sequence #3), and video-video (sequence #4).

### Data Analysis

The primary outcome of this study was the difference in percent scores in the exit and retention exam for students preferring text-based case presentations during the intervention phase compared with those preferring video-based case presentations. Having a total number of 8 ECSs with a free choice, the cutoff for allocation to the video-preference group was set to having chosen the video format at least four times (ie, ≥50% exposure to the video format). According to this, 2 groups of students were compared with each other by means of an independent *t* test. Data are presented as mean (standard deviation) or percentages (n) as appropriate. Significance levels were set to 5%.

Statistical analysis was performed using IBM SPSS Statistics, version 24.00 (SPSS Inc) and GraphPad Prism, version 5.0 (GraphPad Software Inc).

## Results

### Student Recruitment and Characteristics

A total of 100 out of 102 eligible students for study inclusion provided written consent. Of these, 25 students missed at least one study-related formative exam, resulting in a total number of 75 students with complete data for analysis (effective response rate=73.5%). According to their most frequent choice, 52 students were allocated to the text-preferring group and 23 to the video-preferring group. There were no statistical differences between both groups regarding age at entry exam, attendance at intervention ECS, and percent score achieved in exams during the previous term, taking into account the number of points scored by a particular student as well as the maximum of available points for that same student in the preceding term (see Table 1).

### Format Attendance

**Figure 2:**
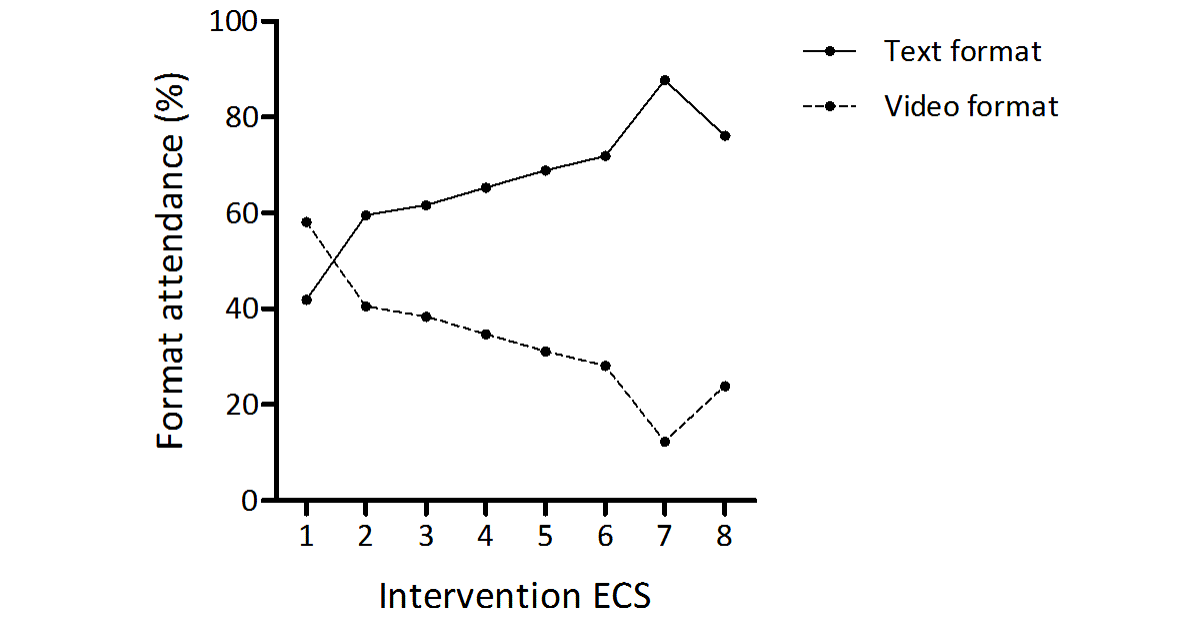
Format attendance. Proportion of students choosing either presentation format during electronic case seminars. ECSs: electronic case seminars.

The proportion of students choosing either format was calculated for each ECS. For text-based ECSs, this proportion ranged from 41.9% (n=31) to 87.7% (n=57), and for video-based ECSs, it ranged from 12.3% (n=9) to 58.1% (n=43; see Figure 2). The number of students preferring text-based over video-based items increased during the intervention phase.

For all items, the predominant learning sequence was *text-text*. The least common learning sequence for all items was *text-video* (see Table 2 for detailed results).

**Table 1:** Characteristics of text- and video-preference groups at entry exam.

Characteristics	Preference for text (n=52), mean (SD)	Preference for video (n=23), mean (SD)	*P* value
Age at entry exam (years)	24.87 (3.40)	24.04 (1.70)	.27
Number of attended intervention electronic case seminars	8.31 (0.64)	8.43 (0.59)	.42
Score achieved in exams of previous semester	82.40 (5.90)	83.50 (7.50)	.56

**Table 2:** Sequences of learning condition. Each item was learned in one of 4 sequences according to students’ choice of presenting format. For study assessment at exit and retention exam, 28 items were assessed in a fixed format listed here.

Item	Sequences of learning condition for each assessment item								Item assessment format
	text-text (#1)		text-video (#2)		video-text (#3)		video-video (#4)		
	Students, n (%)	Mean score at retention exam, %	Students, n (%)	Mean score at retention exam, %	Students, n (%)	Mean score at retention exam, %	Students, n (%)	Mean score at retention exam, %	
1	39 (63)	82	2 (3)	100	5 (8)	40	16 (26)	69	Text-based
2	39 (63)	74	2 (3)	100	5 (8)	80	16 (26)	88	Video-based
3	39 (63)	36	2 (3)	50	5 (8)	80	16 (26)	63	Text-based
4	39 (63)	46	2 (3)	50	5 (8)	40	16 (26)	56	Video-based
5	39 (63)	54	2 (3)	50	5 (8)	60	16 (26)	50	Video-based
6	37 (69)	76	2 (4)	100	9 (17)	89	6 (11)	100	Video-based
7	37 (69)	81	2 (4)	50	9 (17)	89	6 (11)	100	Text-based
8	37 (69)	86	2 (4)	100	9 (17)	89	6 (11)	100	Text-based
9	31 (52)	84	2 (3)	100	10 (17)	90	17 (28)	100	Text-based
10	31 (52)	55	2 (3)	50	10 (17)	70	17 (28)	71	Video-based
11	41 (63)	44	0 (0)	—^a^	16 (25)	50	8 (12)	88	Text-based
12	41 (63)	85	0 (0)	—	16 (25)	75	8 (12)	100	Video-based
13	31 (52)	58	2 (3)	50	10 (17)	70	17 (28)	53	Video-based
14	31 (52)	48	2 (3)	100	10 (17)	30	17 (28)	47	Text-based
15	25 (35)	52	6 (8)	83	19 (26)	53	22 (31)	68	Text-based
16	25 (35)	32	6 (8)	67	19 (26)	16	22 (31)	23	Video-based
17	24 (38)	96	3 (5)	100	21 (33)	100	15 (24)	100	Video-based
18	37 (64)	65	4 (7)	50	7 (12)	86	10 (17)	60	Video-based
19	37 (64)	92	4 (7)	100	7 (12)	100	10 (17)	90	Text-based
20	37 (64)	54	4 (7)	75	7 (12)	57	10 (17)	50	Text-based
21	39 (56)	72	5 (7)	60	7 (10)	43	19 (27)	63	Text-based
22	36 (58)	75	2 (3)	100	8 (13)	63	16 (26)	94	Video-based
23	37 (64)	81	4 (7)	100	7 (12)	86	10 (17)	90	Text-based
24	44 (59)	91	0 (0)	—	0 (0)	—	30 (41)	90	Video-based
25	44 (59)	64	0 (0)	—	0 (0)	—	30 (41)	67	Text-based
26	37 (69)	78	2 (4)	100	9 (17)	100	6 (11)	100	Video-based
27	37 (69)	84	2 (4)	100	9 (17)	89	6 (11)	100	Text-based
28	37 (69)	27	2 (4)	0	9 (17)	67	6 (11)	17	Video-based

^a^Not applicable as no student chose this sequence for this item.

**Figure 3:**
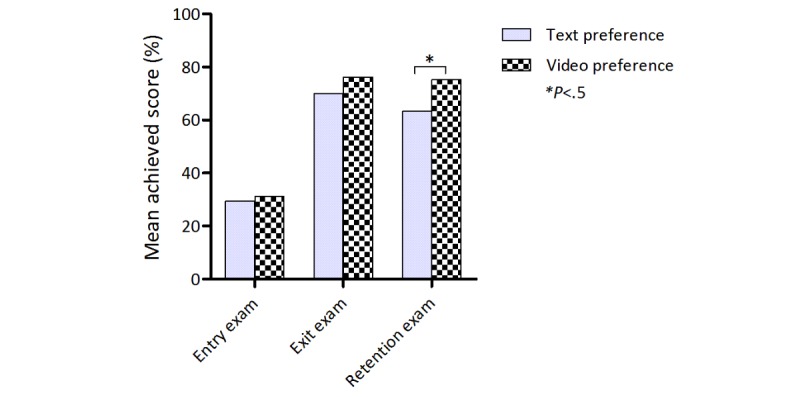
Exam scores. Mean percent scores in the entry, exit, and retention exams for the text-preferring and video-preferring group.

**Figure 4:**
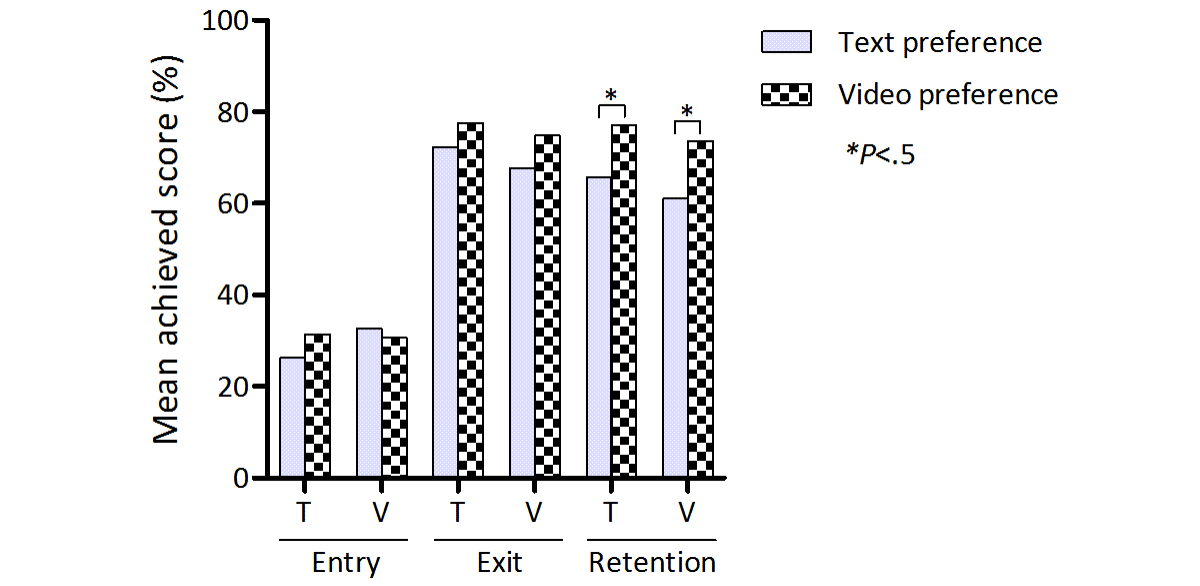
Exam scores by presentation format in the formative exams. Mean percent scores in the entry, exit, and retention exams for the text-preference and video-preference groups. Data are presented as a function of exposure during the intervention phase (column texture) and item format in the formative exams (text vs video). T: text; V: video.

### Learning Outcome

In the entry and exit exam, there was no significant difference in percent scores between students preferring video-based items and students preferring text-based items (entry exam: 31.1% [SD 12.3] vs 29.4% [SD 12.3]; *P*=.59; exit exam: 76.2% [SD 12.6] vs 70.0% [SD 19.0]; *P*=.15). In the retention exam, students who had preferred videos during the intervention phase scored significantly higher than students preferring text-based items (75.3% [SD 16.6] vs 63.4% [SD 20.3]; *P*=.02; see Figure 3).

Exam performance was further analyzed according to the way items were presented in the formative exams. As described above, 14 of the 28 items were displayed as videos whereas the other half were presented in written form. The main effect of preferring videos during the intervention phase persisted, regardless of presentation format in the formative exams (see Figure 4): Mean percent scores in text-based items in the exit exam were 77.6% (SD 14.0; students preferring video) versus 72.4% (SD 20.2; students preferring text; *P*=.26). For video-based items, these figures were 74.8% (SD 12.7) versus 67.6% (SD 20.0); *P*=.11). Differences were significant in the retention test (text-based items: 77.0% (SD 18.8) vs 65.8% (SD 21.2); *P*=.03; video-based items: 73.6% (SD 16.6) vs 61.0% (SD 21.2); *P*=.01).

## Discussion

This study yielded 2 principal findings: First, the presentation format preferences of students changed in favor of the less time-consuming written format over the course of the intervention phase. Second, students preferring the video-based format outperformed students preferring text-based items in the retention exam, regardless of the item presentation format.

### Student Preferences

Several studies reported that students had a positive attitude toward videos for case presentations and that they preferred video- compared with text-based learning [14,15,23,24]. Thus, the current finding of a shift toward text-based items and the fact that almost 70% of enrolled students had to be allocated to the text-preference group is somewhat surprising. However, this finding is in accordance with results from a recently published study reporting a preference for text-based learning material in 65% of undergraduate medical students [25]. A detailed analysis of the differences between the 2 formats seems warranted as they relate to various aspects of the student experience that may well impact on the learning outcome. The most obvious differences relate to time, learner engagement, the amount of context given and the *presence* of virtual patients. With regard to time, the aforementioned study [25] concluded that one of the drawbacks of video use is that it slows down the pace of the seminar and does not allow students to review and critically appraise the presented information. Yet, students acknowledged that videos provide more detailed and contextual information than written material does. In fact, videos provide more complex information.

According to the cognitive load theory [26], medical students in one particular year of undergraduate education can still be regarded as a heterogeneous group of learners. Some may find it easier to deal with more complex material whereas learners lacking experience or exposure to clinical content might be overwhelmed by the wealth of audio-visual information contained in videos [27]. This might be the reason why some students appeared to prefer video-based case presentations at the beginning but switched to the text-based format in the course of the study. In addition, one recent study found that learner engagement was reduced in video-based training compared with other educational approaches [28], and video-based patient cases may even disrupt deep critical thinking [15]. Thus, the provision of more contextual information and a more realistic presence of virtual patients in the learning environment does not guarantee better learning outcomes. A qualitative approach may be warranted to explore learner experience when exposed to video- or text-based material. On the basis of the data collected in this study, we cannot comment on these aspects. Yet, findings in the field of learning in general [29,30] and specifically in medical education [31,32] strongly suggest that learner experience moderates learning outcome.

Another potential explanation for the shift in preferences observed in this study is that working with text-based case histories took less time than working with video-based case histories. In any case, the difference in time-on-task between the 2 preference groups might account for the net finding of superior retention exam performance in students preferring video-based case presentations.

### Learning Outcome

The findings of this study confirm previous results regarding a positive effect of test-enhanced learning on clinical reasoning by using key feature questions for case-based learning [11]. Both study groups achieved a sustained performance gain compared with the entry exam.

The more favorable learning outcome observed in the video-preference group is in concordance with other studies [20]. Notably this advantage was independent of the way items were presented in the formative exams as students preferring video-based case presentations during ECSs also achieved higher scores in retention exam items that were assessed in written form. This is in line with the dual-coding theory which posits that as images and words are processed in different parts of the brain, the use of visualization with sound enhances learning and recall [33]. On the basis of this notion, Kamin et al demonstrated the superiority of video-enhanced learning material for the acquisition of critical thinking [16].

The importance of context for learning outcome was demonstrated over 40 years ago [34], and it could be argued that increased authenticity of the learning environment might help students achieve a better learning outcome. In fact, in a randomized study with 288 medical students, there was no overall advantage for more authentic formats, but in a subanalysis, authors showed that this effect was driven by a strong benefit observed in the top tertile whereas all other students scored fewer points following exposure to the more authentic format [21]. This supports the conclusion that video-based case presentations may only be more effective than text-based presentations for a specific subset of students who may or may not be aware of this.

### Implications and Perspectives

This study adds to the literature in that it helps curriculum planners, medical teachers, and students make informed choices about the design of instructional material. There is a strong rationale for using video-based case presentations combined with key feature questions for teaching clinical reasoning, but it has to be considered that not all students benefit in the same way and at the same time. About one-third of medical students seem to benefit from video-based case presentations. This might be explained by students having an individual preference for audio-visual learning, although other mechanisms cannot be ruled out and should be addressed in future studies. Giving students the opportunity to choose the presentation format they prefer at each single seminar seems to be a reasonable and feasible approach to avoid disadvantages for anyone and to take advantage of the potential of a more authentic format. Furthermore, this would also add up to the described use of mixed methods by being allowed to learn both text and video-based in the course of a curriculum [35]. In the context of computer-based learning, it should not be a huge challenge to implement such formats, and it could help each student use an appropriate format. One important question is how students who did not benefit from the intervention in this study may be helped to capitalize on the merits of repeated testing. One earlier trial suggested that the effectiveness of the method can be enhanced by informing students about the effects of test-enhanced learning [8]. Apart from this, the role of assessments has to be reconsidered especially in the light of recent studies regarding test-enhanced learning and the important role of assessments on students’ learning strategies [36,37]. However, students may need to be briefed about the pros and cons of each format [8]. Ideally, future studies will identify short test instruments providing students with individual feedback regarding the presentation format that is likely to be most beneficial to them. In addition, further studies should explore why the effect of different learning modalities might only become apparent after some time and not directly following exposure to the teaching material.

### Strength and Limitations

To the best of our knowledge this is the first prospective study using case-based key feature questions for teaching clinical reasoning, allowing students to select their individual learning material. The formative exit and retention exams contained both text- and video-based items to minimize potential effects of training to any format. The items themselves referred to relevant problems of general medicine, and the response rate was favorable.

However, this is a monocentric study with a selected group of students as only fourth-year medical students were allowed to participate. Thus, findings of our study are not generalizable to other student groups and subject areas other than general medicine. Regarding ethical aspects, it was not possible to establish a study design without free choice of format as this study was conducted in the official curriculum and there was no way of knowing whether students randomized to either group would be disadvantaged. Hence, self-selection as a potential bias has to be taken into account when interpreting the findings of this study. Furthermore, we did not collect any quantitative or qualitative data on student experience during ECSs. However, as differences between the 2 presentation formats in terms of time, engagement, context, and the *presence* of virtual patients may impact on learning outcome, these aspects should be addressed in future studies. Finally, it was technically not feasible to measure the time individual students spent on every single item. However, it can be assumed that reading was less time consuming than watching the respective video.

### Conclusions

Although about two-thirds of medical students preferred text-based case presentations, those students who self-selected to work on video-based presentations achieved better long-term retention of procedural knowledge as assessed with key feature questions. As clinical reasoning is one of the most complex but important objective in medical education, more research is needed to identify the most effective approach to teaching and learning related skills.

## Appendix

Multimedia Appendix 1 Presentation format. Screenshot of a text- (A) and a video-based (B) format. For reasons of privacy, faces are blurred out.

## Abbreviations

ECS: electronic case seminar
