# Supplementary figures and images for: The Impact of Medical Students’ Individual Teaching Format Choice on the Learning Outcome Related to Clinical Reasoning
Source: JMIR Med Educ. 2019 Jul 22;5(2):e13386. doi: 10.2196/13386 (PMC6681636; doi:10.2196/13386)

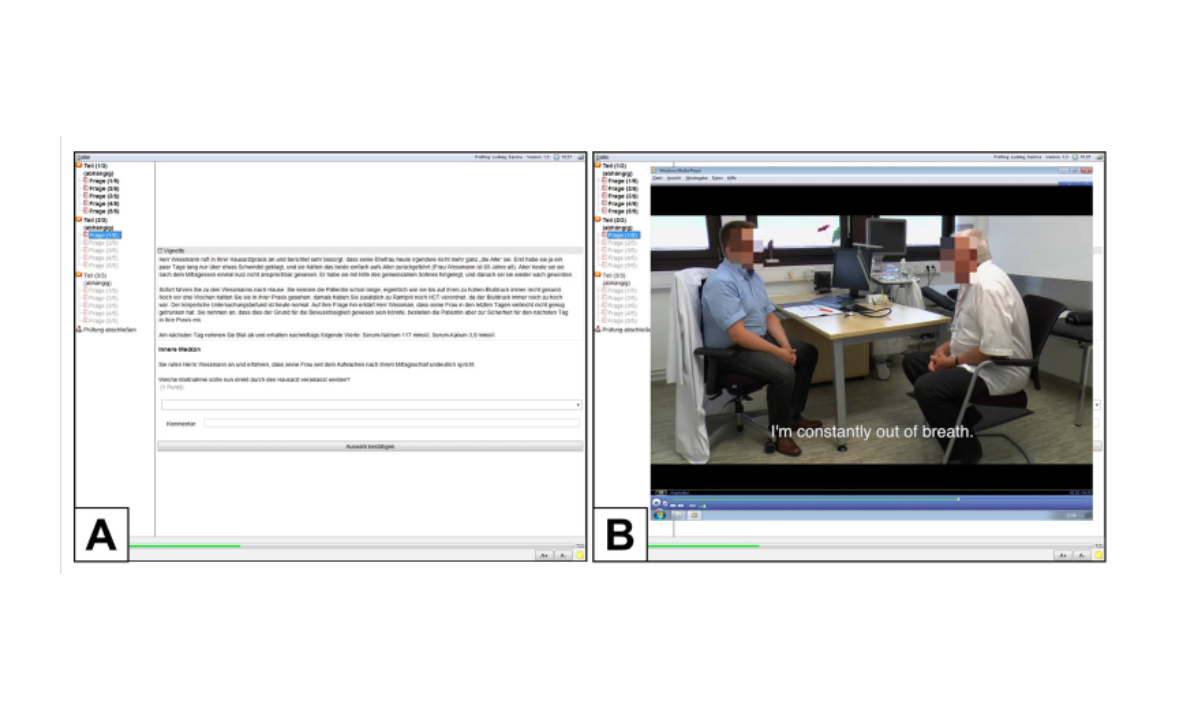

Supplement: Multimedia Appendix 1 [file mededu_v5i2e13386_app1.png]
